# The In Vitro Inhibitory Effect of Selected Asteraceae Plants on Pancreatic Lipase Followed by Phenolic Content Identification through Liquid Chromatography High Resolution Mass Spectrometry (LC-HRMS)

**DOI:** 10.3390/ijms231911204

**Published:** 2022-09-23

**Authors:** Aristeidis S. Tsagkaris, Anna Louckova, Tereza Jaegerova, Viola Tokarova, Jana Hajslova

**Affiliations:** 1 Department of Food Analysis and Nutrition, Faculty of Food and Biochemical Technology, University of Chemistry and Technology Prague, Technická 5, Prague 6–Dejvice, 166 28 Prague, Czech Republic; 2 Department of Chemical Engineering, Faculty of Chemical Engineering, University of Chemistry and Technology Prague, Technická 5, Prague 6–Dejvice, 166 28 Prague, Czech Republic

**Keywords:** enzyme assay, in vitro testing, bioprospecting, obesity, suspect screening, phytochemicals, polyphenols, ultra-high-performance liquid chromatography hybrid quadrupole time-of-flight mass spectrometry, metabolomics

## Abstract

Pancreatic lipase (PNLIP, EC 3.1.1.3) plays a pivotal role in the digestion of dietary lipids, a metabolic pathway directly related to obesity. One of the effective strategies in obesity treatment is the inhibition of PNLIP, which is possible to be achieved by specific phenolic compounds occurring in high abundance in some plants. In this study, a multidisciplinary approach is presented investigating the PNLIP inhibitory effect of 33 plants belonging in the Asteraceae botanical family. In the first stage of the study, a rapid and cost-efficient PNLIP assay in a 96-microwell plate format was developed and important parameters were optimized, e.g., the enzyme substrate. Upon PNLIP assay optimization, aqueous and dichloromethane Asteraceae plant extracts were tested and a cut-off inhibition level was set to further analyze only the samples with a significant inhibitory effect (inhibitory rate > 40%), using an ultra-high-performance liquid chromatography hybrid quadrupole time-of-flight mass spectrometry (UHPLC-q-TOF-MS) method. Specifically, a metabolomic suspect screening was performed and 69 phenolic compounds were tentatively identified, including phenolic acids, flavonoids, flavonoid-3-O-glycosides, and flavonoid-7-O-glycosides, amongst others. In the case of aqueous extracts, phytochemicals known for inducing PNLIP inhibitory effect, e.g., compounds containing galloyl molecules or caffeoylquinic acids, were monitored in *Chrysanthemum morifolium*, *Grindella camporum* and *Hieracium pilosella* extracts. All in all, the presented approach combines in vitro bioactivity measurements to high-end metabolomics to identify phenolic compounds with potential medicinal and/or dietary applications.

## 1. Introduction

**Figure 1:**
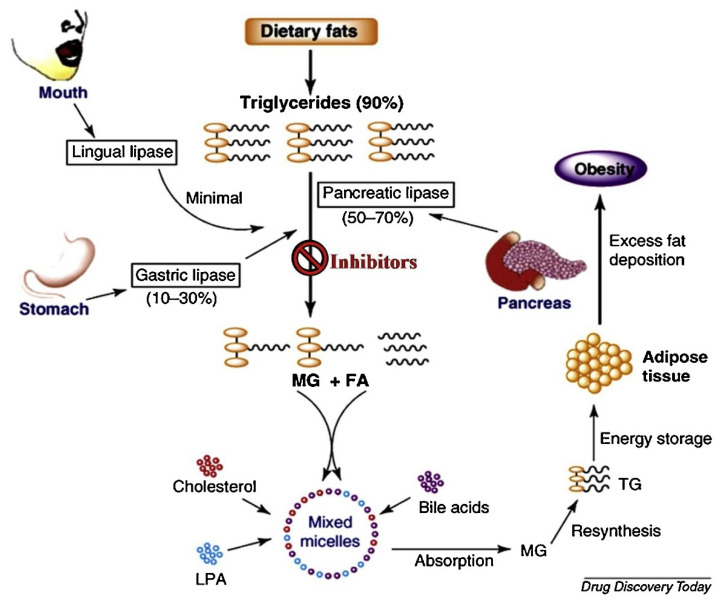
Lipids metabolism in humans and the role of PNLIP in these metabolic pathways [1], under a Creative Commons license.

Pancreatic lipase (PNLIP) (EC 3.1.1.3), an important lipolytic enzyme secreted by the pancreas into the digestive tract, is primarily responsible for the hydrolysis and absorption of dietary lipids from the intestines. As shown in Figure 1, triacylglycerols, representing the most abundant component of dietary lipids, are hydrolyzed (up to 70%) to monoacylglycerols and free fatty acids by the action of PNLIP. The impact of this lipid metabolic pathway can be of utmost importance in obesity treatment. Specifically, the inhibition of PNLIP activity may result in reduced synthesis of adipose tissue preventing from excessive fat deposition [1]. Considering the obesity associated risks and its high prevalence in the western population [2], PNLIP activity inhibition resulting in reduced fat metabolism can significantly impact obesity treatment. Up to date, there is only one drug in clinical use acting as PNLIP inhibitor, namely, “orlistat”, that was originally derived from the natural compound lipstatin [3,4]. The main advantage of using orlistat as an anti-obesity agent is that it does not affect the central nervous system (CNS), a major drawback of other medications resulting, sometimes, in psychological effects [5]. Therefore, it is necessary to find more natural sources of PNLIP inhibitors, i.e., phenolic acids or polyphenols, contained in plant extracts and study their prospect to be implemented in anti-obesity medications.

Focusing on phenolic compounds, they are secondary metabolites contained in relatively high concentrations in plant tissues and contribute to plant defense against UV-radiation or aggression against pathogens [6]. Importantly, phenolics have shown proven bioactivity, including antioxidant, cardiovascular protective and anti-inflammatory properties, amongst others [7]. In terms of anti-obesity properties, there is ongoing evidence that phenolic compounds inhibit PNLIP activity. Interestingly, such analytes are abundantly found in various plant sources, i.e., gallic acid or quercetin have been studied to provide insight on the inhibition mechanism [8]. In fact, there have been various plant matrices reported with PNLIP inhibitory potency, for example, berry plants [9] or tea extracts [10]. Among them, plants belonging to the Asteraceae botanical family have attracted significant attention by the research community [11,12], focusing on testing extracts of differing polarity as well as different plant parts. The reason behind the warm interest on Asteraceae plants is that they are widely available all over the world with over 2500 plant species. Additionally, they have proven antimicrobial activity [13] and inhibit the activity of enzymes with important biochemical functions [12,14]. Characteristic examples of such plants are lettuce (*Lactuca sativa*), artichoke (*Cynara cardunculus*) (which are both popular vegetables) as well as medicinal plants, such as chamomile (*Anthemis nobilis*) [15] or dandelion (*Taraxacum officinale*) [16]. Last but not least, to monitor PNLIP activity, there are various analytical approaches mostly using spectroscopic detection, for example, absorbance or fluorescence, as it comprehensively discussed in [17].

In this study, an in-house PNLIP spectrophotometric assay was developed and optimized to identify Asteraceae plants that can effectively inhibit PNLIP activity and potentially find medicinal applications. During the PNLIP assay optimization, special focus was paid on the standardization of the analytical signal acquisition since this has been a significant bottleneck in the field of bioactivity studies [18]. The optimized assay was used to monitor the inhibitory effect of 72 Asteraceae plant extracts on PNLIP and those attained a significant inhibitory effect (>40% inhibition rate) were analyzed on an ultra-high-performance liquid chromatography hybrid quadrupole time-of-flight mass spectrometry (UHPLC-q-TOF-MS) system. A suspect screening workflow was applied and in-house spectral database was constructed containing 240 Asteraceae phytochemicals. In this way, a tentative identification of the tested extracts composition was achieved indicating their bioprospecting potential and highlighting the prevalence of polyphenols in the Asteraceae extracts.

## 2. Results and Discussion

Identifying natural compounds with anti-PNLIP effects, a strategy that can find use as an alternative medicine in obesity treatment, is of indispensable importance, as the obese population is constantly increasing [19]. In addition, the development of medication that does not impact the central nervous system is of great interest to avoid psychological side-effects [4]. To date, only one such drug, orlistat, is available in clinical use underlining the need to find more potent natural sources [3]. To investigate the Asteraceae plant extract inhibitory effect, we developed a robust and sensitive PNLIP assay. Within this context, optimization experiments were performed evaluating the effect of various parameters on assay performance. In detail, the following parameters (see Section 2.1 and Section 2.2) were tested: (i) enzyme substrate, (ii) enzyme concentration, (iii) effect of organic solvents on PNLIP activity, (iv) enzyme-sample incubation time and (v) color production time. Afterwards, the optimized PNLIP was utilized to rapidly screen the inhibitory effect of the Asteraceae extracts. A 40% inhibitory rate was applied as a cut-off level (see Section 2.3) and the extracts exceeding this value were further investigated. Firstly, serial extract dilutions were prepared to evaluate the dose-response effect on PNLIP activity. Afterwards, these extracts were analyzed using a UHPLC-q-TOF-MS instrument to tentatively identify their phenolic composition based on a suspect screening workflow (see Section 2.4).

### 2.1. Testing of Different Substrates Resulting in Coloured and Fluorescent Products

Developing a PNLIP assay is a rather challenging analytical task due to the solubility of hydrophobic substrates in aqueous buffers, which are necessary to retain enzyme activity. One possible solution on that is the use of emulsions or reversed micelles which significantly increases the method complexity and analysis duration [20]. In this study, to deliver a rapid and simple solution, three synthetic substrates (nitrophenyl acetate, indoxyl acetate, 4-methylumbelliferone) were tested to identify which fits the purpose. Important to note is that all three substrates were readily dissolved in DMSO omitting the need for emulsification as dimethyl sulfoxide (DMSO) and phosphate buffer saline (PBS) are miscible. The colorimetric nitrophenyl acetate (NPA) reaction was the fastest providing a sufficient signal (detectable absorbance change) within 15 min followed by 4-methylumbelliferone (4-MUO) hydrolysis, which provided a fluorescent signal within 30 min. The attained response was different in these two cases. Specifically, increasing NPA concentration resulted in an increased attained signal whilst the 4-MUO acquired signal decreased for concentrations higher than 5 mM (see Figure S1 in the Supplementary Materials, Section S1). In terms of substrate price per gram (Table 1), purchasing NPA cost from 2.5 to 50 times less (in comparison to indoxyl acetate and 4-MUO, respectively), indicating that it is the best option to deliver a low-cost analytical method. Besides featuring a low price, NPA also provided a sufficient colorimetric response and for these reasons was selected as the assay substrate and solely used during the next stages of method optimization (see Section 3.2).

### 2.2. PNLIP Optimization Using NPA as the Substrate

**Figure 2:**
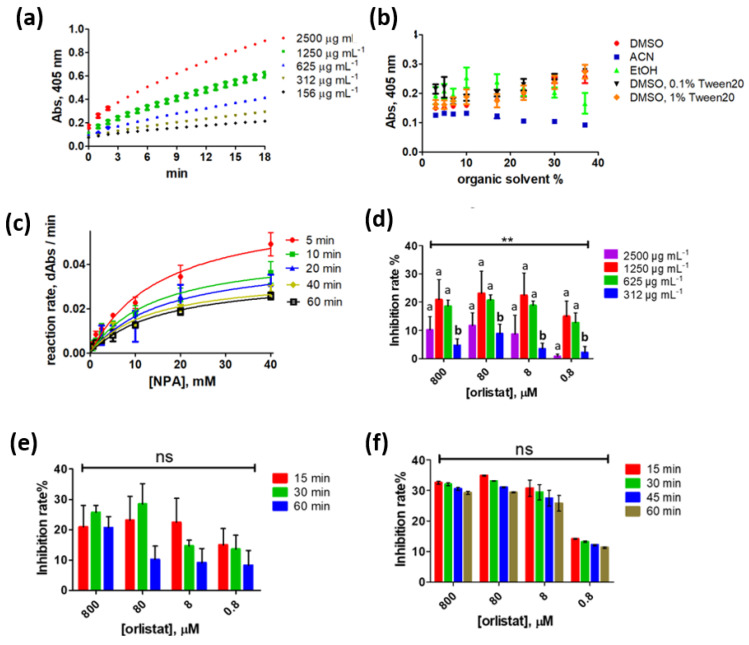
The investigated assay parameters: (**a**) monitored absorbance at 405 nm vs. time (min), *n* = 4 replicates per level, (**b**) Michaelis–Menten kinetics calculated at 5 different time intervals, *n* = 6 replicates per level (**c**) effect of organic solvent type and tween 20 (surface active compound) on PNLIP. PBS as buffer (pH = 7.4, 1250 µg mL^−1^ PNLIP), (**d**) effect of PNLIP concentration on the inhibition rate, (**e**) effect of incubation time on the inhibition rate (**f**) effect of end point on the inhibition rate. Each column represents the mean value (*n* = 4) and the error bars represent the standard deviation in each case. Kruskal–Wallis test followed was performed to reveal statistically significant differences at the 95% confidence level; **: *p*-value < 0.01; ns: non-significant. Different letters indicate significant differences among the groups based on the Dunn’s multiple comparison test.

Upon selecting NPA as the assay substrate, a comprehensive method optimization was performed to achieve optimum analytical performance. To begin with, 1250 µg mL^−1^ PNLIP provided a sufficient signal (Figure 2a) and this concentration was used to calculate the Michaelis–Menten constant (Km) and the enzyme reaction max velocity (Vmax) using seven different NPA levels (0.625, 1.25, 2.5, 5, 10, 20 and 40 mM). It was found that depending on the selected end-point (Figure 2b and Table S1 in the Supplementary Materials, Section S2) Km mean value fluctuated from 14 to 17 mM. Nevertheless, considering that high substrate concentrations can result in enzyme activity inhibition [21] decreasing the hydrolysis rate, 10 mM of NPA were used as the substrate concentration. Km is an important parameter representing the substrate concentration (on this occasion NPA) at which the reaction velocity is equal to half the maximal velocity of the reaction (1/2 Vmax). In terms of enzyme reaction velocity, a decreased rate was monitored at a longer end-point, which can be considered reasonable based on the temporal substrate consumption. In other words, signal production is faster in the initial reaction stages, a characteristic assuring that rapid screening can be achieved based on NPA hydrolysis.

Identifying the PNLIP tolerance toward organic solvents was of outmost importance as enzyme activity can be negatively impacted due to protein denaturation. Among the tested solvents (Figure 2c), DMSO aqueous solutions (up to 40% DMSO in PBS) enhanced the acquired signal indicating that DMSO can be used without worrying about potential loss of enzyme activity. Similar behavior was noticed for DMSO aqueous solutions containing minor amounts of tween-20 (0.1% and 1%), a surfactant helping with enzyme solubility. In contrast to DMSO, acetonitrile (ACN, another aprotic solvent) strongly decreased the acquired signal, indicating that extracts prepared in ACN cannot be measured by the assay. In the case of the protic ethanol, signal enhancement was noticed up to 10% ethanol (EtOH) in PBS followed by a signal constant decrease. The noticed signal enhancement (due to a better substrate solubility) and decrease (due to protein native structure alteration) are considered reasonable as they are in line with previous findings [22].

To optimize the assay detectability, 4 orlistat concentrations (0.8, 8, 80, 800 µM) were measured under different conditions and the attained inhibition rate was used as a detectability indicator. The effect of: (i) PNLIP concentration (Figure 2d), (ii) sample-enzyme incubation time (Figure 2e), and (iii) colored product development time (i.e., end-point, Figure 2f) on the inhibition rate was sequentially monitored. Firstly, the highest inhibition rate (>40% in the range 8–800 µM) was noticed when using 1250 µg mL^−1^ PNLIP and this enzyme concentration was selected providing enough signal (Figure 2a) and thus sufficient detectability. Both similar [23] and higher [24] orlistat inhibition rates have been reported and such differences can be related to the enzyme manufacturer, enzyme purity (in this study a type II PNLIP was used), and always expected interlaboratory differences. Afterwards, the incubation period of the sample with PNLIP was investigated. This period is necessary to permit the enzyme to interact with a potential inhibitor. Although a 30 min incubation time sometimes provided higher inhibition rates (Figure 2e, at 800 and 80 µM levels), such differences were not statistically significant according to the performed non-parametric Kruskal–Wallis test (at a 95% confidence level). Considering that among the study goals was to deliver a rapid screening method and the statistically insignificant differences noticed, the 15 min incubation period was selected. In line with the sample-enzyme incubation period, measuring after the shortest color development time (15 min) resulted in the highest inhibition rate. In terms of method duration, our in-house PNLIP assay achieved similar or even faster [25,26] results in comparison to other studies. All in all, a 30 min total analysis time was enough to sensitively monitor orlistat inhibitory effect on PNLIP and the achieved optimized conditions were applied to monitor the anti-PNLIP effect of the selected plants (see Section 2.3).

### 2.3. Screening of the Inhibitory Effect of the Studied Plants on PNLIP

**Figure 3:**
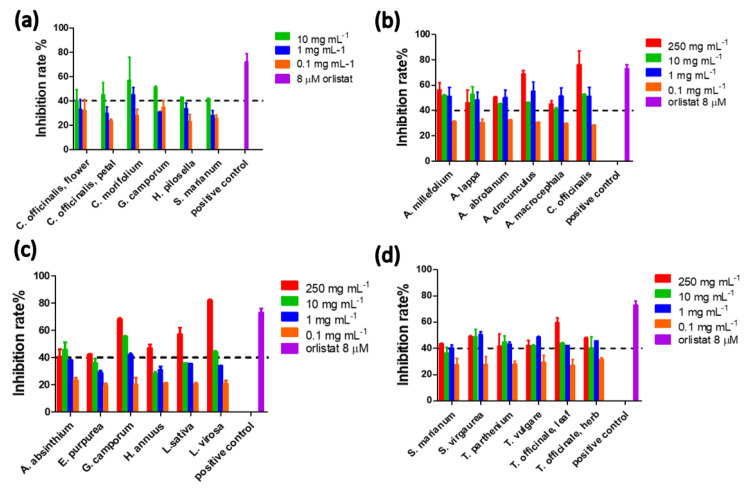
Monitored inhibitory effect on PNLIP activity induced by serial dilutions of the selected Asteraceae plant extracts. (**a**) Aqueous and (**b**–**d**) DCM extracts of the Asteraceae plants exceeding the 40% cut-off inhibition rate.

Extracts from 33 Asteraceae plants were monitored using the PNLIP in-house assay (Table 2). In the case of the aqueous extracts (10 mg mL^−1^), eight samples induced an inhibition rate higher than 40% and for these cases serial dilutions (from 10 to 0.1 mg mL^−1^) were performed resulting in a respective decrease in the monitored PNLIP inhibitory effect (Figure 3). *G. camporum,* known as grindelia herb showed the highest PNLIP inhibition, specifically 51%, followed by chamomile (*A. nobilis*, 45%) and milk thistle seed (*S. marianum)* extracts. In the case of dichloromethane (DCM) extracts, half of the samples exceeded the cut-off level, a result that may be related to the higher crude non-polar extract concentration (250 mg mL^−1^), which was possible due to the better solubility of plant components in DCM. In this occasion, the inhibitory rate was mostly stable for most of the extracts (Figure 3b–d) in the range of 250 to 1 mg mL^−1^ plant DCM extract. In other words, there was not a clear dose-response effect. The inhibitory effect was clearly reduced only at 0.1 mg mL^−1^ of DCM extract. Interestingly, strong anti-PNLIP effect was found for the DCM extracts, specifically, an 82% of inhibition was monitored for wild lettuce (*L. virosa*), a plant known for its high content in hydroxyderivatives of cinnamic acid [27] (e.g., caffeic, ferulic, synapic and p-coumaric acids), compounds with known PNLIP inhibition. A 76% of inhibition was attained by marigold petal (*C. officinalis*) extracts, a plant with reported medicinal use around the globe and significant inhibitory effect towards PNLIP [28,29]. Overall, there has been significant interest towards the Asteraceae family plants and their effect on PNLIP with genera *Artemisia* [30], *Cynara* [31], *Eupatorium* [30], *Inula* [30], featuring results similar to our study.

### 2.4. Tentative Identification of the Tested Extract Metabolites through Suspect Screening

To begin with, 52 probable structures were identified in the aqueous extracts (Table 3), specifically, phenolic acids, flavonoids, and their glucosides, e.g., flavonoid-3-O-glycosides or flavonoid-7-O-glycosides. In the case of DCM extracts, less compounds were identified in comparison to the aqueous extracts, specifically, 17 phenolic compounds (Table 4). To further identify non-polar compounds in the DCM extracts, it will be necessary to update the developed suspect list or apply a non-targeted screening to achieve a better mapping of their phytochemical composition. Other potential components into the DCM extracts can be long-chain fatty acids, alcohols, alkanes, esters, or triterpenoids. Worthy to notice is that medium polarity analytes were identified in both aqueous and DCM extracts of different plants, such as apigenin or quercetin. Following the identification criteria proposed by Schymanski et al. [32], all the analytes could be recognized at a level 2 identification, or in other words, the reported results represent the probable analyte structures. A level 2 identification means that it was possible to propose an exact structure using different evidence, namely MS and MS/MS data as well as comparison towards spectral libraries. To further confirm the presence of these analytes, it would have been necessary to buy the respective analytical standards (if available). Nevertheless, the high number of identified analytes would significantly increase the cost of such a purchase and for this reason a higher identification level was not possible.

**Figure 4:**
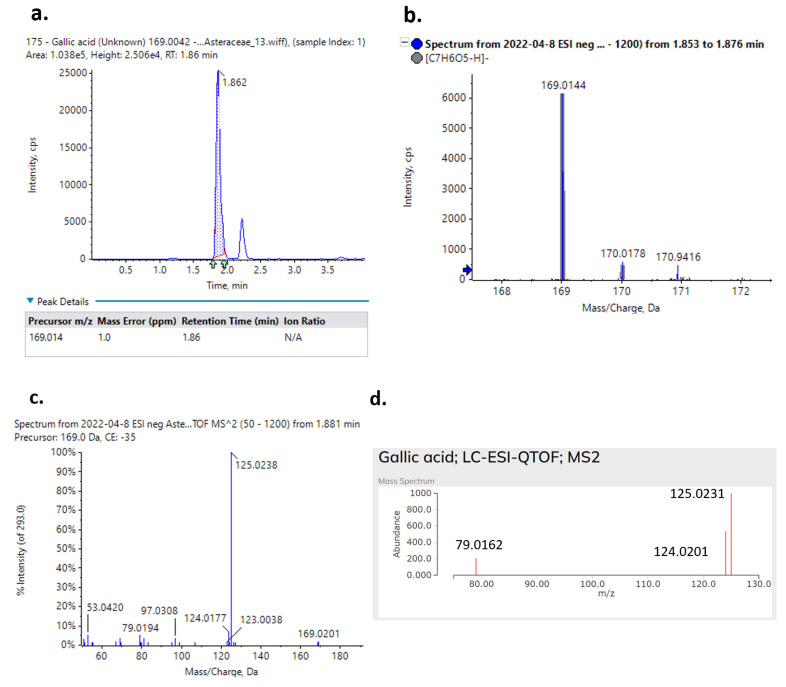
Gallic acid identification process. (**a**) The monitored XIC chromatogram. (**b**) The attained MS and (**c**) MS/MS spectra. (**d**) The available online MS/MS spectrum available on https://massbank.eu/MassBank/RecordDisplay?id=PR308148&dsn=RIKEN, last accessed 2 August 2022.

In all cases, excellent mass accuracy was achieved, typically lower than 2 ppm. The identification was achieved by comparing the experimental MS/MS fragments with MS/MS spectra found in mass spectral libraries or in other published studies. To indicatively showcase the applied workflow, the identification process of gallic acid in *C. morifolium* is presented (Figure 4). Firstly, the extracted ion chromatogram (XIC) of 169.0140 corresponding to the pseudomolecular ion [M-H]^−^ was displayed (Figure 4a) featuring a very low mass error, approximately 1 ppm, and a high ion intensity (approximately 25,000). The peak recorded with a retention time equal to 2.2 min was an isobaric compound (a compound with the same nominal mass but with a different molecular formula) that was not investigated as the ion intensity was lower than the set cut-off limit (<1000, see Section 3.7 for more information). Then, the MS spectrum (Figure 4b) of the mass feature of interest was evaluated to assure that is not a result of an in-source fragment by any other m/z existing in the mass spectrum. Finally, the obtained MS/MS spectrum (Figure 4c) was compared towards a record (Figure 4d) in the MassBank of North America (https://massbank.eu/MassBank/RecordDisplay?id=PR308148&dsn=RIKEN, last accessed 2 August 2022) and confirmed the presence of almost identical fragments for the mass 169.0140. On both occasions, a C18 column was used and a difference of 0.3 min (1.86 min in this study and 2.04 min on the MassBank record) was noticed between the two measurements providing further evidence of the presence of gallic acid in the extract. Importantly, gallic acid (detected in *C. morifolium*, *G. camporum*, *H. pilosella* extracts) is a compound with a proven PNLIP inhibitory effect. Gallic acid and other galloyl moiety compounds, induced a competitive mode of inhibition against PNLIP [33]. In fact, we identified epigallocatechin 3-O-gallate or catechin 3-O-gallate isomers monitored in *C. officinalis* extracts from flowers and petals. In a previous study, these compounds were found to molecularly interact with PNLIP by changing the active site and preventing substrate access [34]. Interestingly, a correlation between the number of galloyl moieties on the flavanol molecule and PNLIP inhibition rate was found [35], indicating the potential of such compounds to find a medicinal application.

Focusing on other identified compounds with proven anti-PNLIP effect, apigenin and its glucosides (e.g., apigenin-7-glucoside) were found in the *C. morifolium*, *G. camporum*, *H. pilosella* extracts. Nevertheless, such flavones are considered to have a lower inhibitory potency than the molecules with galloyl moieties [33]. Another group of compounds identified in the measured polar extracts (Table 3) was isomers of the dicaffeoylquinic and caffeoylquinic acids. This comes in line with other findings suggesting that Asteraceae species contain high concentrations of caffeoylquinic acids [12] compounds with proven bioactivity including PNLIP inhibition. Similar to the galloyl phytochemicals, a competitive inhibition mode was also reported for these analytes [36], which were able to bind and interact with the catalytic triad of Ser153, His264, and Asp177 at the PNLIP active site. The glycosylated flavonoid linarin was detected in *C. morifolium*. This analyte is a characteristic metabolite of the Asteraceae plants and has demonstrated diverse bioactivity [37], including PNLIP and acetylcholinesterase (another hydrolase of significant biochemical importance) inhibition.

**Figure 5:**
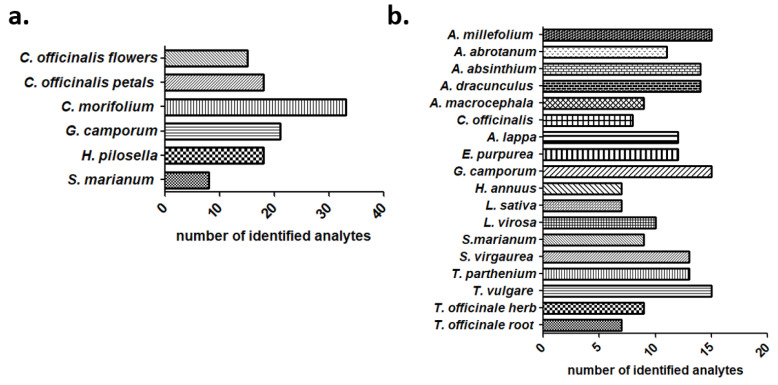
Number of identified phytochemicals per extract (**a**) for aqueous and (**b**) for DCM extracts. The attained results were acquired through the described suspect screening workflow.

Lastly, besides the total number of identified compounds, it is also important to consider how many analytes were identified in each of the extracts based on the metabolomic suspect screening. The highest number of identified compounds was found in *C. morifolium* aqueous extracts (33 analytes) followed by *G. camporum* (21 analytes) (Figure 5a), whilst in the case of DCM extracts most samples had approximately 10 identified compounds (Figure 5b). The XIC chromatograms of the *C. morifolium* aqueous extract (Figure S2 in the Supplementary Materials, Section S3) and *A. millefolium* DCM extract (Figure S3 in the Supplementary Materials, Section S3) are presented in the Supplementary Materials to indicatively showcase the acquired peaks shape.

## 3. Materials and Methods

### 3.1. Chemicals

PBS tablets, tween-20, type II porcine PNLIP, IDA (purity > 95%), 4-MUO (purity > 95%), NPA (purity > 98%), ammonium formate (purity > 99.9%), DMSO (purity 99%), EtOH (purity 99%), ACN (purity 99%), and orlistat (purity 98%) were supplied by Sigma Aldrich (Prague, Czech Republic). Microplates (96-well format) were bought by Gama Group (České Budějovice, Czech Republic). HPLC methanol (MeOH, purity > 99.9%), isopropanol (IPA, purity > 99.9%), and formic acid (FA, purity > 99.9%) were purchased from Honeywell Riedel-de Haën (Prague, Czech Republic).

### 3.2. PNLIP Assay Substrate Selection

**Figure 6:**
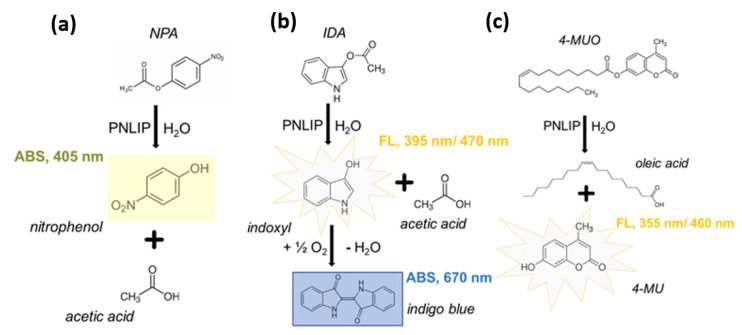
In vitro hydrolysis of (**a**) NPA, (**b**) IDA, and (**c**) 4−MUO by PNLIP, resulting in both colored and fluorescent products.

One of the most critical steps in enzyme activity assays is the selection of an appropriate substrate providing a sufficient analytical signal. Within this study, to achieve a rapid PNLIP activity screening, three synthetic artificial substrates were used, namely NPA, IDA and 4-MUO [17]. In every case, the assays were adjusted to a 96-microwell plate format and the detailed protocols are provided in the Supplementary Materials (see Section S4). Interestingly, the selected substrates provide both colored (NPA and IDA) and fluorescent products (IDA and 4-MUO) (Figure 6) permitting a critical comparison among the tested reactions to pick the most suitable substrate for this study (see Section 2.1). Finally, absorbance measurements were performed in an Epoch BioTek reader (Winooski, VT, USA) and fluorescence measurements in an Infinite^®^ 200 PRO reader (Tecan, Switzerland).

### 3.3. In-Vitro PNLIP Assay

After selecting the most suitable substrate (see Section 2.1) further investigation of critical parameters was performed, e.g., PNLIP concentration, incubation time, tolerance against organic solvents, and parameters affecting assay detectability (see Section 2.2). During method optimization, to identify statistically significant differences among the tested groups (e.g., different enzyme concentration or incubation period), the non-parametric Kruskal–Wallis test, followed by Dunn’s multiple comparison test, was performed at a significance level, α = 0.05 using GraphPad prism 5.0 software (San Diego, CA, USA). Based on these experiments, the attained optimal conditions were identified and are reported here. In detail, a PNLIP solution (1250 μg mL^−1^) in PBS containing 0.1% tween-20 was prepared in a 50 mL centrifugal plastic tube. After solution preparation, centrifugation at 10,000 revolutions per minute (rpm) (Rotina 380R, Hettich, Tuttlingen, Germany) for 2 min was performed to reduce insoluble impurities contained in the dried PNLIP powder. Afterwards, 80 μL PNLIP were incubated with 10 μL of a sample in DMSO for 15 min. The sample could be (i) a plant extract, (ii) blank DMSO as a negative control, or (iii) an orlistat DMSO solution in a specific concentration as a positive control. When the incubation period was completed, 10 μL of 10 mM NPA in DMSO were added and the absorbance was measured at 405 nm after 15 min. The experiments were performed during three independent days, in triplicate (each day), and the data were pooled.

### 3.4. Tested Plant Extracts and Extract Preparation

The Asteraceae plant extracts (see Table S2 for sample details in the Supplementary Materials, Section S5), were purchased and prepared by Caitheness Biotechnologies (Leicester, UK), a certified provider of plant materials. Briefly, based on the provider documentation, the aqueous extracts were prepared by drying the fresh material using a desiccator (at 37 °C for 12–18 h). A total of 25 g of dried crushed material was added to 250 mL boiling distilled water and steeped overnight in the dark at 4 °C. The suspension was filtered using Whatman number 1 chromatography paper. Filtrates were lyophilized and the freeze-dried powder was stored at −80 °C. Lastly, the freeze-dried powder was resuspended at 10 mg mL^−1^ in 100% DMSO and insoluble material was discarded. In the case of the non-polar extracts, DCM was used as the extractant. Similarly, the extracts were prepared from dry fresh material using a desiccator (at 37 °C for 12–18 h). A total of 10 g of crushed plant material was added to 100 mL DCM at room temperature and steeped overnight in the dark at 4 °C. A rotary evaporator was used to remove the majority of DCM and the residual DCM was evaporated using a gentle nitrogen stem. Finally, the dried product was resuspended in 40 mL DMSO resulting in a final extract concentration of 250 mg mL^−1^ and insoluble material was discarded.

Upon arrival in our laboratory, the extracts were stored in −80 °C using 96-microwell plates (see Figure S4 in the Supplementary Materials, Section S5). Before analysis, the frozen extracts were left to condition to room temperature for 2 h and then subjected to the procedure described in Section 3.3. Considering that non-specific PNLIP inhibition is possible due to matrix components, e.g., extracted colored pigments, a cut-off level of significant inhibition rate equal to 40% was set following the strategy of Slanc et al. [38]. When a concentrated extract (10 mg mL^−1^ for aqueous and 250 mg mL^−1^ for DCM extracts) induced an inhibition of at least 40% or higher, then serial dilutions were performed to reach a concentration of 10, 1, and 0.1 mg mL^−1^, and the dose–response effect was monitored. In addition, such extracts (>40% inhibition rate) were further analyzed using UHPLC-q-TOF-MS to tentatively identify their composition based on a suspect screening workflow (see Section 3.7).

### 3.5. In Vitro PNLIP Assay Data Processing and Handling

The color of the tested plant extracts highly varied due to their composition (colored compounds, such as chlorophylls, carotenoids, anthocyanins) indicating the chance of potential spectral interferences when measuring absorbance. Besides plant extract components, the enzyme substrate (in this case NPA) can also potentially contribute to the attained signal, due to autoxidation, resulting in an additional error source. Therefore, it is necessary to minimize the effects of these interferences by using appropriate sample and reagent blanks for raw data correction [18]. For every performed assay, the raw absorbance data were blank-corrected as it is described in the formulas included in the supplementary materials (See Section S6 in the Supplementary Materials, Formulas (S1)–(S3)). PNLIP inhibition was expressed as inhibition rate% and calculated using the Formula (S4) (see Section S6 in Supplementary Materials). For each sample, the same measurement was performed in two independent days and the inhibition data were pooled (*n* = 4 in total per sample). Finally, the figures provided in Section 3 were designed using GraphPad prism 5.0 software (San Diego, CA, USA).

### 3.6. UHPLC-q-TOF-MS Analysis

When a tested extract attained more than 40% of inhibition rate, it was diluted 10-times in methanol (reaching a concentration of 1 mg mL^−1^ and 25 mg mL^−1^ in the case of aqueous and DCM extracts, respectively) to avoid injecting high matrix content into the chromatographic system that could significantly impact its performance and was chromatographically analyzed. The UHPLC-q-TOF-MS analysis was performed on a DionexUltiMate 3000 chromatograph (Thermo Fisher Scientific, Waltham, MA, USA) coupled with a TripleTOF™ 6600 (SCIEX, Vaughan, ON, Canada) mass spectrometer based on the conditions of a recently published study of our group [39] with slight modifications. To control the LC-part of the analyzer, the Chromeleon TM (Thermo Fisher Scientific, Waltham, MA, USA) software was utilized whilst the MS part was controlled through the Analyst 1.7.1 TF software (SCIEX, Concord, ON, Canada). In detail, the separation of polar extracts was carried out in an HSS T3 (2.1 × 100 mm, 1.8 µm) analytical column at 45 °C. The mobile phase consisted of A: deionized water with 5 mM ammonium formate and 0.1% formic acid and B: methanol with 5 mM ammonium formate and 0.1% formic acid. The gradient used was: 0–5 min (5% B), 5–11 min (5–50% B), 11–18 min (50–100% B), 18–19 (100% B). The separation of the non-polar extracts was carried out on a BEH C18 (2.1 × 100 mm, 1.7 µm) analytical column at 45 °C. The mobile phase consisted of A: mixture of deionized water with methanol (95:5), with 5 mM ammonium formate and 0.1% formic acid and B: mixture of 2-propanol, methanol, and deionized water (65:30:5), with 5 mM ammonium formate and 0.1% formic acid. The following gradient was utilized: 0–1 min (10% B), 1–14 min (10% B), 14–19 min (10–100% B), 19–19.1 min (100% B). The injection volume was 2 µL in both cases and the flow rate was 0.4 mL min^−1^. Mass spectra were obtained in both positive and negative ionization mode with electrospray ionization. The acquisition mode was programmed to obtain spectra in full MS mode and to obtain MS/MS spectra. The electrospray ionization was performed using the following parameters: capillary temperature was 500/450 °C; capillary voltage was +5000 V/−4000 V; collision energy was 35 eV (±15 eV).

### 3.7. Suspect Screening Workflow to Tentatively Identify the Selected Extract Composition

To apply a suspect screening workflow [40,41], a database of secondary metabolites reported in the Asteraceae family was created. To achieve that, a review of the recent scientific literature on the analysis of Asteraceae species and their phytochemical composition was performed obtaining a compound list. For these compounds, a manual search was performed and the following information was added (if possible): (i) alternative names, (ii) molecular formula, (iii) chemical class, (iv) plant source, (v) chemical identifiers (CAS, PubChem, ChemSpider), and (vi) reported biological activity. In total, the database contained 196 Asteraceae metabolites and 44 compounds with reported inhibitory effect originating from various plant sources. The database is provided as an Excel file in the Supplementary Materials and used references are reported here [42,43,44,45,46,47,48,49,50,51,52,53,54,55,56,57,58,59,60,61,62]. To evaluate the results generated based on the suspect list screening, the SCIEX OS (version 1.5.0.23389, Vaughan, ON, Canada) software was used. The criteria for compound identification were: (i) the exact mass, (ii) mass error (<5 ppm), (iii) isotope profile, (iv) peak area (>2000) and ion intensity (>1000) threshold, and (v) conformity of mass fragmentation spectra with spectra on online mass spectra libraries (www.mzcloud.com, www.pubchem.com, www.massbank.eu, accessed on 2 August 2022) and other publications cited in the suspect list.

## 4. Conclusions

An in-house PNLIP assay was developed and optimized achieving high throughput (up to 96 measurements per run), low cost (estimated less than one EUR per microwell plate), and rapid results (30 min run time). It was proven that the developed assay can be satisfactorily used to evaluate the inhibitory effect of plant extracts toward PNLIP. Nevertheless, considering that various matrix compounds may inhibit PNLIP activity, the fragmentation of the crude extracts is necessary in a follow-up study, which is already planned. Among the tested aqueous extracts, the grindelia herb showed the highest PNLIP inhibition (51%), while in terms of DCM extracts, wild lettuce achieved the highest inhibition (82%). In general, a higher inhibition rate was monitored for the DCM extracts, which can be related to either higher concentration in comparison to the aqueous extracts or to lipophilic unknown compounds contained in the DCM extracts. In addition, the coexistence of bioactive compounds in some extracts indicate the potential of synergistic effects that could be of interest to study in vitro to attain a better understanding of the interaction between PNLIP and the phytochemical “cocktails”. Following the samples in vitro investigation, the extracts with a significant inhibitory effect were further chromatographically analyzed to tentatively identify their composition. The presence of various bioactive phytochemicals was monitored using a suspect screening workflow based on high resolution mass spectrometry (HRMS). Importantly, some of the proposed analytes contained in the tested extracts were compounds with reported PNLIP inhibitory effect. Overall, the present study combined a simple bioanalytical assay with high end metabolomic analysis to identify the presence of polyphenols and phenolic compounds in the tested Asteraceae extracts and highlight their potential in bioprospecting studies. Work is underway to develop more enzyme assays with important biochemical functions, such as α-glucosidase or tyrosinase assays, aiming to comprehensively monitor the bioactivity profile of promising plant materials and showcase their potential medicinal and/or nutritional applications.

## Figures and Tables

**Table 1:** Critical comparison of the application of NPA, indoxyl acetate (IDA), and 4-MUO as the substrate. The same PNLIP concentration was used (1250 μg mL^−1^) in all cases to provide comparable results.

Assay Characteristic	NPA	IDA		4-MUO
optical detection	absorbance, yellow product (405 nm)	absorbance, blue product (670 nm)	fluorescence, λexc = 395 nm & λem = 470 nm	fluorescence, λexc = 355 nm & λem = 460 nm
substrate concentration range	1.25–20 mM		0.625–10 mM	
substrate cost per g *	60 EUR	150 EUR		3000 EUR
total assay time **	30 min	75 min	55 min	45 min

* The cost per g of substrate was estimated based on the price of the respective chemicals needed for each analysis (based on the Merck website for the Czech market, https://www.sigmaaldrich.com/CZ/en, last accessed on 18 August 2022); ** This duration includes a 15 min incubation period prior to enzyme reaction product detection.

**Table 2:** Mean attained PNLIP inhibition rate% by the aqueous (10 mg mL^−1^, *n* = 4) and DCM (250 mg mL^−1^, *n* = 4) extracts obtained from plants of the Asteraceae family.

Species	Common Name	Plant Part	Aqueous Extract Inhibition Rate (%)	SD	DCM Extract Inhibition Rate (%)	SD
*Achillea millefolium*	Yarrow	Leaf	30	3.7	56	6.3
*Arctium lappa*	Burdock	Leaf	29	1.4	46	10
*Arctium lappa*	Burdock	Root	16	4.00	39	8.3
*Artemisia abrotanum*	Southernwood herb	Leaf	20	1.4	50	0.90
*Artemisia absinthium*	Wormwood	Aerial part	28	0.799	41	5.5
*Artemisia annua*	Sweet wormwood (Qing Hao)	Stem	11	20	33	1.1
*Artemisia dracunculus*	Tarragon	Leaf	2.3	20	69	2.6
*Anthemis nobilis*	Chamomile	Flower	43	0.88	45	1.6
*Artemisia vulgaris*	Mugwort herb	Aerial part	34	16	39	1.4
*Atractylodes macrocephala*	Atractylodes	Rhizome	22	35	45	2.5
*Calendula officinalis*	Marigold	Petal	45	10	76	25
*Calendula officinalis*	Marigold	Flower	40	9.4	36	0.46
*Cichorium intybus*	Chicory root	Root	27	0.21	35	3.2
*Cnicus benedictus*	Holythistle	Aerial part	21	0.46	39	1.5
*Cynara cardunculus*	Artichoke	Leaf of stem	28	0.39	34	2.2
*Eclipta alba*	Bhringaraj root	Root	20	0.47	22	11
*Echinacea angustifolia*	Narrow-leaved purple coneflower	Root	27	6.2	36	4.3
*Echinacea purpurea*	Purple coneflower	Root	29	0.26	42	0.15
*Eupatorium perfoliatum*	Boneset	Leaf	23	7.4	27	15
*Eupatorium purpureum*	Gravel root	Root	19	3.4	4.8	3.1
*Grindelia camporum*	Grindelia herb	Aerial part	51	1.1	68	0.65
*Helianthus annuus*	Sunflower seed	Seed	0	0	47	2.5
*Hieracium pilosella*	Mousear, hawkweed	Aerial part	43	0.22	35	6.5
*Chrysanthemum morifolium*	Chrysanthemum flowers	Flower	57	19	29	10
*Inula helenium*	Elecampane Root	Root	6.6	4.1	38	5.7
*Lactuca sativa*	Lettuce	Leaf	6	7.1	57	5.0
*Lactuca virosa*	Wild lettuce	Leaf	11	13	82	0.71
*Matricaria recutita*	German chamomile	Flower	38	0.17	29	0.41
*Silybum marianum*	Milk thistle seed	Seed	43	0.25	43	0.55
*Solidago virgaurea*	Golden rod	Aerial part	29	5.2	49	0.40
*Stevia rebaudiana*	Stevia leaf	Leaf	24	0.57	4.8	2.1
*Tanacetum parthenium*	Feverfew herb	Aerial part	26	2.9	42	9.4
*Tanacetum vulgare*	Tansy herb	Aerial part	31	1.5	42	3.8
*Taraxacum officinale*	Dandelion herb	Leaf	38	12	60	3.6
*Taraxacum officinale*	Dandelion root	Root	12	2.09	48	0.79
*Tussilago farfara*	Coltsfoot	Aerial part	33	0.13	31	13

**Table 3:** Proposed phytochemicals contained in the selected aqueous extracts through metabolomic suspect screening. All the proposed compounds are identified in a level 2 confidence.

Class	Compounds	Detected Ion	Molecular Formula	Measured *m*/*z*	Δ ppm	tR (min)	Fragment Ions (*m*/*z*)	Tentatively Identified in
flavanols	(−)-Catechin 3-O-gallate/Epicatechin 3-O-gallate	[M-H]^−^	C_22_H_18_O_10_	441.0824	−0.8	4.77	125.0243, 169.0139, 245.0842, 289.0723	*C. officinalis* flowers and petals
	(−)-Epigallocatechin 3-O-gallate/(−)-Gallocatechin 3-O-gallate	[M-H]^−^	C_22_H_18_O_11_	457.0769	−1.6	3.99	125.0245, 169.0149, 193.0123, 292.8134	*C. officinalis* flowers and petals
flavanones	Eriocitrin	[M-H]^−^	C_27_H_32_O_15_	595.1666	−0.4	5.13	151.0032, 287.0556	*C. morifolium*
	Eriodictyol	[M-H]^−^	C_15_H_12_O_6_	287.0559	−0.9	5.14	107.0170, 135.0449, 151.0083, 287.0560	*C. morifolium*
	Eriodictyol 7-O-glucoside	[M-H]^−^	C_21_H_22_O_11_	449.1093	0.7	4.98	107.0132, 135.0450, 151.0031, 175.0023, 287.0561	*C. morifolium*
flavones	Apigenin	[M-H]^−^	C_15_H_10_O_5_	269.0461	2.1	6.72	117.0351, 151.007, 269.0469	*C. morifolium, H. pilosella*
	Apigenin 7-O-D-glucoronide	[M-H]^−^	C_21_H_18_O_11_	445.0779	0.5	6.1	269.0527	*C. morifolium, G. camporum, H. pilosella*
	Apigenin 7-O-glucoside	[M-H]^−^	C_21_H_20_O_10_	431.098	−0.7	6.18	268.0377, 269.0456	*C. morifolium, G. camporum, H. pilosella*
	Apigenin 7-O-rutinoside	[M-H]^−^	C_27_H_30_O_14_	577.1565	0.4	6.08	269.0456	*C. morifolium, H. pilosella*
	Aromadendrin	[M-H]^−^	C_15_H_12_O_6_	289.0692	−5.1	5.85	107.0468, 121.0259, 149.0206, 153.0156	*S. marianum*
	Chrysoeriol/Hispidulin/Diosmetin	[M-H]^−^	C_16_H_12_O_6_	299.0561	−0.1	6.82	284.0239, 285.0280	*C. morifolium, G. camporum*
	Diosmetin 7-O-6″-acetylglucoside	[M-H]^−^	C_24_H_24_O_12_	503.1192	−0.7	6.81	284.0327, 299.0568	*C. morifolium*
	Diosmetin 7-O-glucoronide	[M-H]^−^	C_22_H_20_O_12_	475.0881	−0.2	6.13	284.0329, 299.0571	*G. camporum*
	Diosmetin 7-O-glucoside	[M-H]^−^	C_22_H_22_O_11_	461.1088	−0.3	6.35	284.0366, 299.0585	*C. morifolium*
	Linarin	[M-H]^−^	C_28_H_32_O_14_	591.1728	1.4	7.25	268.0395, 283.0628	*C. morifolium*
	Vicenin 2	[M-H]^−^	C_27_H_30_O_15_	593.1507	−0.7	4.6	353.0680, 383.0799, 473.1068, 593.1549	*C. officinalis* petals, *C. morifolium, G. camporum, H. pilosella*
flavonols	Astragalin/Luteolin 3′-glucoside/Luteolin 7-O-glucoside/Trifolin	[M-H]^−^	C_21_H_20_O_11_	447.0937	1.0	5.73	284.0343, 285.0425, 447.0969	*C. morifolium, H. pilosella*
	Isoquercetin/Hyperoside/Quercetin 3-O-glucoside/Quercetin 7-O-galactoside/Quercetin 7-O-glucoside/Spiraein	[M-H]^−^	C_21_H_20_O_12_	463.0889	1.4	5.86	255.0272, 271.0319, 300.0327, 301.0401, 463.0844	*C. morifolium, S. marianum*
	Isorhamnetin 3-O-glucoside	[M-H]^−^	C_22_H_22_O_12_	477.1041	0.4	6.4	271.0172, 285.0453, 314.0433, 315.0442, 477.1034	*C. morifolium*
	Kaempferol/Luteolin	[M-H]^−^	C_15_H_10_O_6_	285.0405	0.1	6.41	107.0141, 133.0298, 151.0032, 175.0399, 285.0460	*C. morifolium*
	Kaempferol 3-glucuronide/Luteolin 7-O-glucoronide	[M-H]^−^	C_21_H_18_O_12_	461.0725	−0.1	5.17	285.043	*H. pilosella*
	Luteolin 7-O-(6″-acetylglucoside)	[M-H]^−^	C_23_H_22_O_12_	489.1041	0.4	6.28	284.0329, 285.0398	*C. officinalis* petals, *C. morifolium, H. pilosella*
	Luteolin 7-O-(6″-malonylglucoside)	[M-H]^−^	C_24_H_22_O_14_	533.0937	0.1	6.28	284.0323, 285.0401, 489.1043	*C. morifolium, H. pilosella*
	Luteolin 7-O-rutinoside/Nicotiflorin	[M-H]^−^	C_27_H_30_O_15_	593.1512	0.1	5.65	285.0408, 593.1519	*C. officinalis* flowers and petals, *C. morifolium, 267*
	Quercetin	[M-H]^−^	C_15_H_10_O_7_	301.0352	−0.7	7.06	63.0259, 65.0031, 83.0122, 108.0236, 134.0361, 145.0322, 149.0603, 151.003, 301.0001	*C. morifolium, G. camporum*
	Quercetin 3-O-(6″-acetyl-glucoside)	[M-H]^−^	C_23_H_22_O_13_	505.0987	−0.2	5.94	271.0228, 300.0290, 301.0356	*C. officinalis* flowers and petals
	Quercetin 3-O-(6″-malonylglucoside)	[M-H]^−^	C_24_H_22_O_15_	549.0905	3.5	6.02	300.0253, 301.0368	*C. officinalis* flowers and petals, *C. morifolium*
	Quercetin 3-O-glucoronide	[M-H]^−^	C_21_H_18_O_13_	477.0673	−0.3	5.12	301.0351	*C. morifolium*
	Rutin	[M-H]^−^	C_27_H_30_O_16_	609.1463	0.3	5.38	300.0277, 301.0354, 609.1475	*C. officinalis* flowers and petals
O-methylated flavone	Eupatilin/Nevadensin	[M-H]^−^	C_18_H_16_O_7_	343.0824	0.28	8.55	298.0113, 313.0346, 328.0580, 343.1569	*C. morifolium, G. camporum*
O-methylated flavonol	Centaureidin	[M-H]^−^	C_18_H_16_O_8_	361.0908	−2.8	7.95	285.0390, 303.0511, 328.0582, 345.0631, 361.0914	*C. morifolium, G. camporum*
O-methylated isoflavone	Acacetin/Biochanin A/Genkwanin	[M-H]^−^	C_16_H_12_O_5_	283.0615	1.2	7.66	268.0371	*C. morifolium*
dihydroflavonols	Taxifolin	[M-H]^−^	C_15_H_12_O_7_	303.0513	1.0	5.13	57.0342, 125.0250, 150.0315, 175.0395, 285.0389	*S. marianum*
phenolic acid	1.3-dicaffeoylquinic acid/1.5-di-O-Caffeoylquinic acid	[M-H]^−^	C_25_H_24_O_12_	515.1246	4.8	5.09	135.0446, 179.0350, 191.0561, 353.0895	*H. pilosella*
	1.4-dicaffeoyl quinic acid/3.4-Dicaffeoylquinic acid/3.5-Dicaffeoylquinic acid/4.5-Dicaffeoylquinic acid	[M-H]^−^	C_25_H_24_O_12_	515.1193	−0.4	5.13	179.0359, 191.0560, 353.0867	*C. officinalis* flowers and petals, *C. morifolium, G. camporum, H. pilosella*
	1-O-caffeoylquinic acid/3-O-caffeoylquinic acid/4-O-caffeoylquinic acid	[M-H]^−^	C_16_H_18_O_9_	353.0879	0.2	3.99	191.0563	*C. officinalis flowers and petals, C. morifolium, G. camporum, H. pilosella, S. marianum*
	Caffeic acid	[M-H]^−^	C_9_H_8_O_4_	179.0348	−0.8	4.3	134.0343, 135.0460	*C. officinalis* flowers and petals, *C. morifolium, G. camporum, H. pilosella*
	Gallic acid	[M-H]^−^	C_7_H_6_O_5_	169.0144	1.2	1.86	51.0229, 79.0185, 124.0130, 125.0253	*Calendula officinalis* petals, *C. morifolium, G. camporum, H. pilosella*
	m-Coumaric acid/o-Coumaric acid/p-Coumaric acid	[M-H]^−^	C_9_H_8_O_3_	163.0399	−0.7	5	93.0335, 119.0493	*G. camporum*
	Quinic acid	[M-H]^−^	C_7_H_12_O_6_	191.0564	1.5	0.7	85.0293, 93.0334, 99.0463, 127.0404, 191.0548	*C. officinalis* flowers and petals, *C. morifolium, G. camporum, H. pilosella, S. marianum*
	Syringic Acid	[M-H]^−^	C_9_H_10_O_5_	197.0455	−0.5	4.39	89.0047, 123. 0089	*C. officinalis* flowers and petals
	Vanillic Acid	[M-H]^−^	C8H8O4	167.035	0.3	4.37	108.0211, 152.0153	*C. officinalis* flowers, *G. camporum*
phenolic aldehyde	Protocatechualdehyde	[M-H]^−^	C_7_H_6_O_3_	137.0245	0.5	3.37	108.0204, 109.0283, 119.0137, 136.0161, 137.0232	*C. officinalis flowers, C. morifolium, G. camporum, H. pilosella, S. marianum*
flavonolignans	isosilybin A/isosilybin B/silybin A/silybin B/silydianin	[M-H]^−^	C_25_H_22_O_10_	481.1143	0.6	7.11	125.0245, 152.0112, 178.9968, 180.0065, 301.0361, 481.1141	*S. marianum*
	silychristin	[M-H]^−^	C_25_H_22_O_10_	481.1143	0.6	6.01	125.0240, 151.0029, 178.9984, 325.0713	*S. marianum*
hydroxycoumarins	scopoletin	[M-H]^−^	C_10_H_8_O_4_	191.0351	0.8	3.7	104.0284, 120.0221, 148.0153	*C. officinalis* flowers and petals, *G. camporum*

**Table 4:** Proposed phytochemicals contained in the selected DCM extracts through metabolomic suspect screening. All the proposed compounds are identified in a level 2 confidence.

Class	Compound	Detected Ion	Molecular Formula	Measured *m*/*z*	Δ ppm	tR (min)	Fragment Ions (*m*/*z*)	Tentatively Identified in
flavanones	Naringenin	[M-H]^−^	C_15_H_12_O_5_	271.0611	−0.3	4.56	107.0154, 119.0511, 271.0597	*A. millefolium, A. abrotanum, A. absinthium, A. dracunculus, A. macrocephala, C. officinalis petals, A. lappa leaf, E.purpurea, G. camporum, S. marianum, S. virgaurea, T. parthenium, T. vulgare*
	Eriodictyol	[M-H]^−^	C_15_H_12_O_6_	287.0558	−1.2	3.79	107.0129, 135.0442, 151.0026, 287.0558	*A. dracunculus, A. lappa leaf, E.purpurea*
flavones	Apigenin	[M-H]^−^	C_15_H_10_O_5_	269.0453	−0.9	5.1	117.0337, 151.0037, 269.0443	*A. millefolium, A. absinthium, A. dracunculus, A. macrocephala, A. lappa leaf, E.purpurea, G. camporum, L. sativa, L. virosa, T. parthenium, T. vulgare, T. officinale herb*
	Apigenin 7-O-glucoside	[M-H]^−^	C_21_H_20_O_10_	431.0976	−1.72	3.2	268.0354, 431.0946	*A. millefolium*
	Chrysoeriol/Hispidulin/Diosmetin	[M-H]^−^	C_16_H_12_O_6_	299.056	−0.30	4.9	227.0366, 256.0363, 284.0308	*A. millefolium, A. absinthium, A. dracunculus, A. macrocephala, A. lappa leaf, E.purpurea, G. camporum, S. virgaurea, T. parthenium, T. vulgare*
flavonolignans	isosilybin A/isosilybin B/silybin A/silybin B/silydianin	[M-H]^−^	C_25_H_22_O_10_	481.1133	−1.48	4.5	125.0238, 152.0124, 178.9981, 180.0058, 273.0404, 301.0362, 481.1176	*S. marianum*
flavonols	Kaempferol/Luteolin	[M-H]^−^	C_15_H_10_O_6_	285.0404	−0.07	4.4	107.0160, 133.0297, 151.0065, 175.0406, 285.0428	*A. millefolium, E.purpurea, T. vulgare*
	Isorhamnetin	[M-H]^−^	C_16_H_12_O_7_	315.0507	−1.02	4.3	227.0322, 243.0332, 283.0360, 300.0283, 315.0528	*A. absinthium, A. dracunculus, T. vulgare*
	Quercetin	[M-H]^−^	C_15_H_10_O_7_	301.0358	1.41	4.6	63.0243, 65.0031, 83.0138, 108.0221, 134.0384, 149.0601, 151.0030, 301.0732	*A. dracunculus*
hydroxycoumarins	umbelliferone	[M-H]^−^	C_9_H_6_O_3_	161.0243	−0.80	1.8	133.0288, 161.0243	*A. millefolium, A. abrotanum, A. absinthium, A. dracunculus, A. lappa leaf, G. camporum, L. virosa, S. virgaurea, T. parthenium, T. vulgare*
	scopoletin	[M-H]^−^	C_10_H_8_O_4_	191.0353	1.44	1.8	120.0205, 148.0166, 191.0283	*A. abrotanum, A. absinthium, C. officinalis petals, G. camporum, S. virgaurea*
isoflavonoids	Formononetin	[M-H]^−^	C_16_H_12_O_4_	267.0662	−0.43	5.6	135.0087, 195.0461, 223.0430, 252.0440	*A. millefolium, A. abrotanum, A. absinthium, G. camporum, H. annuus, T. parthenium, T. vulgare, T. officinale herb, T. officinale root*
O-methylated flavone	Eupatilin/Nevadensin	[M-H]^−^	C_18_H_16_O_7_	343.0817	−1.79	5.8	313.0331, 328.0562, 343.0828	*A. millefolium, A. abrotanum, A. absinthium, G. camporum, T. parthenium, T. vulgare, T. officinale herb*
O-methylated isoflavone	Acacetin/Biochanin A/Genkwanin	[M-H]^−^	C_16_H_12_O_5_	283.0612	0.07	6.5	268.0375	*A. millefolium, A. abrotanum, A. absinthium, A. dracunculus, A. macrocephala, A. lappa leaf, E.purpurea, G. camporum, L. virosa, S. virgaurea, T. parthenium, T. vulgare, T. officinale herb, T. officinale root*
phenolic aldehyde	Protocatechualdehyde	[M-H]^−^	C_7_H_6_O_3_	137.0243	−0.82	1.06	108.0220, 109.0315, 136.0169, 137.0237	*E.purpurea*

## Data Availability

Data are available upon reasonable request. Please contact the corresponding author.

## Supplementary Materials

The following supporting information can be downloaded at: https://www.mdpi.com/article/10.3390/ijms231911204/s1.

## References

[1] Liu T.-T., Liu X.-T., Chen Q.-X., Shi Y. (2020). Lipase Inhibitors for Obesity: A Review. Biomed. Pharmacother..

[2] Endalifer M.L., Diress G. (2020). Epidemiology, Predisposing Factors, Biomarkers, and Prevention Mechanism of Obesity: A Systematic Review. J. Obes..

[3] Kumar A., Chauhan S. (2021). Pancreatic lipase inhibitors: The road voyaged and successes. Life Sci..

[4] Müller T.D., Blüher M., Tschöp M.H., DiMarchi R.D. (2022). Anti-obesity drug discovery: Advances and challenges. Nat. Rev. Drug Discov..

[5] Coulter A.A., Rebello C.J., Greenway F.L. (2018). Centrally acting agents for obesity: Past, present, and future. Drugs.

[6] Mukherjee P.K., Mukherjee P.K. (2019). Chapter 20—Phyto-Pharmaceuticals, Nutraceuticals and Their Evaluation.

[7] Luca S.V., Macovei I., Bujor A., Miron A., Skalicka-Woźniak K., Aprotosoaie A.C., Trifan A. (2020). Bioactivity of dietary polyphenols: The role of metabolites. Crit. Rev. Food Sci. Nutr..

[8] Martinez-Gonzalez A.I., Alvarez-Parrilla E., Díaz-Sánchez Á.G., de la Rosa L.A., Núñez-Gastélum J.A., Vazquez-Flores A.A., Gonzalez-Aguilar G.A. (2017). In vitro inhibition of pancreatic lipase by polyphenols: A kinetic, fluorescence spectroscopy and molecular docking study. Food Technol. Biotechnol..

[9] McDougall G.J., Kulkarni N.N., Stewart D. (2009). Berry polyphenols inhibit pancreatic lipase activity in vitro. Food Chem..

[10] Gondoin A., Grussu D., Stewart D., McDougall G.J. (2010). White and green tea polyphenols inhibit pancreatic lipase in vitro. Food Res. Int..

[11] Vitalini S., Garzoli S., Sisto F., Pezzani R., Argentieri M.P., Scarafoni A., Ciappellano S., Zorzan M., Capraro J., Collazuol D. (2022). Digestive and gastroprotective effects of *Achillea erba-rotta* subsp. *moschata* (Wulfen) I. Richardson (syn. *A. moschata* Wulfen) (Asteraceae): From traditional uses to preclinical studies. J. Ethnopharmacol..

[12] Spínola V., Castilho P.C. (2017). Evaluation of Asteraceae herbal extracts in the management of diabetes and obesity. Contribution of caffeoylquinic acids on the inhibition of digestive enzymes activity and formation of advanced glycation end-products (in vitro). Phytochemistry.

[13] Rolnik A., Olas B. (2021). The Plants of the Asteraceae Family as Agents in the Protection of Human Health. Int. J. Mol. Sci..

[14] Koc S., Isgor B.S., Isgor Y.G., Shomali Moghaddam N., Yildirim O. (2015). The potential medicinal value of plants from Asteraceae family with antioxidant defense enzymes as biological targets. Pharm. Biol..

[15] Akbar S. (2020). *Chamaemelum nobile* (L.) all. (asteraceae/aompositae). Handbook of 200 Medicinal Plants.

[16] Grauso L., Emrick S., de Falco B., Lanzotti V., Bonanomi G. (2019). Common dandelion: A review of its botanical, phytochemical and pharmacological profiles. Phytochem. Rev..

[17] Pohanka M. (2019). Biosensors and bioassays based on lipases, principles and applications, a review. Molecules.

[18] Lankatillake C., Luo S., Flavel M., Lenon G.B., Gill H., Huynh T., Dias D.A. (2021). Screening natural product extracts for potential enzyme inhibitors: Protocols, and the standardisation of the usage of blanks in α-amylase, α-glucosidase and lipase assays. Plant Methods.

[19] Ng M., Fleming T., Robinson M., Thomson B., Graetz N., Margono C., Mullany E.C., Biryukov S., Abbafati C., Abera S.F. (2014). Global, regional, and national prevalence of overweight and obesity in children and adults during 1980–2013: A systematic analysis for the Global Burden of Disease Study. Lancet.

[20] Park J.-Y., Ha J., Choi Y., Chang P.-S., Park K.-M. (2021). Optimization of Spectrophotometric and Fluorometric Assays Using Alternative Substrates for the High-Throughput Screening of Lipase Activity. J. Chem..

[21] Aslanzadeh S., Ishola M.M., Richards T., Taherzadeh M.J. (2014). An overview of existing individual unit operations. Biorefineries.

[22] Kamal M.Z., Yedavalli P., Deshmukh M.V., Rao N.M. (2013). Lipase in aqueous-polar organic solvents: Activity, structure, and stability. Protein Sci..

[23] Kim G.-N., Shin M.-R., Shin S.H., Lee A.R., Lee J.Y., Seo B.-I., Kim M.Y., Kim T.H., Noh J.S., Rhee M.H. (2016). Study of Antiobesity Effect through Inhibition of Pancreatic Lipase Activity of *Diospyros kaki* Fruit and *Citrus unshiu* Peel. Biomed Res. Int..

[24] Zhang J., Kang M.-J., Kim M.-J., Kim M.-E., Song J.-H., Lee Y.-M., Kim J.-I. (2008). Pancreatic lipase inhibitory activity of taraxacum officinale in vitro and in vivo. Nutr. Res. Pract..

[25] Hou X.-D., Ge G.-B., Weng Z.-M., Dai Z.-R., Leng Y.-H., Ding L.-L., Jin L.-L., Yu Y., Cao Y.-F., Hou J. (2018). Natural constituents from Cortex Mori Radicis as new pancreatic lipase inhibitors. Bioorg. Chem..

[26] Dechakhamphu A., Wongchum N. (2022). Investigation of the kinetic properties of *Phyllanthus chamaepeuce* Ridl. extracts for the inhibition of pancreatic lipase activity. J. Herb. Med..

[27] Stojakowska A., Malarz J., Szewczyk A., Kisiel W. (2012). Caffeic acid derivatives from a hairy root culture of *Lactuca virosa*. Acta Physiol. Plant..

[28] Hernández-Saavedra D., Pérez-Ramírez I.F., Ramos-Gómez M., Mendoza-Díaz S., Loarca-Pina G., Reynoso-Camacho R. (2016). Phytochemical characterization and effect of *Calendula officinalis*, *Hypericum perforatum*, and *Salvia officinalis* infusions on obesity-associated cardiovascular risk. Med. Chem. Res..

[29] Zaki A., Ashour A., Mira A., Kishikawa A., Nakagawa T., Zhu Q., Shimizu K. (2016). Biological activities of oleanolic acid derivatives from *Calendula officinalis* seeds. Phyther. Res..

[30] Sharma N., Sharma V.K., Seo S.-Y. (2005). Screening of some medicinal plants for anti-lipase activity. J. Ethnopharmacol..

[31] Danış O., Ogan A., Anbar D., Basak Yuce D., Demir S., Salan U. (2015). Inhibition of pancreatic lipase by culinary plant extracts. Int. J. Plant Biol. Res..

[32] Schymanski E.L., Jeon J., Gulde R., Fenner K., Ruff M., Singer H.P., Hollender J. (2014). Identifying small molecules via high resolution mass spectrometry: Communicating confidence. Environ. Sci. Technol..

[33] Rahim A.T.M.A., Takahashi Y., Yamaki K. (2015). Mode of pancreatic lipase inhibition activity in vitro by some flavonoids and non-flavonoid polyphenols. Food Res. Int..

[34] Wang S., Sun Z., Dong S., Liu Y., Liu Y. (2014). Molecular interactions between (−)-epigallocatechin gallate analogs and pancreatic lipase. PLoS ONE.

[35] Nakai M., Fukui Y., Asami S., Toyoda-Ono Y., Iwashita T., Shibata H., Mitsunaga T., Hashimoto F., Kiso Y. (2005). Inhibitory Effects of Oolong Tea Polyphenols on Pancreatic Lipase in Vitro. J. Agric. Food Chem..

[36] Hu B., Cui F., Yin F., Zeng X., Sun Y., Li Y. (2015). Caffeoylquinic acids competitively inhibit pancreatic lipase through binding to the catalytic triad. Int. J. Biol. Macromol..

[37] Mottaghipisheh J., Taghrir H., Boveiri Dehsheikh A., Zomorodian K., Irajie C., Mahmoodi Sourestani M., Iraji A. (2021). Linarin, a Glycosylated Flavonoid, with Potential Therapeutic Attributes: A Comprehensive Review. Pharm..

[38] Slanc P., Doljak B., Kreft S., Lunder M., Janeš D., Štrukelj B. (2009). Screening of selected food and medicinal plant extracts for pancreatic lipase inhibition. Phyther. Res. Int. J. Devoted to Pharmacol. Toxicol. Eval. Nat. Prod. Deriv..

[39] Navratilova K., Hurkova K., Hrbek V., Uttl L., Tomaniova M., Valli E., Hajslova J. (2022). Metabolic fingerprinting strategy: Investigation of markers for the detection of extra virgin olive oil adulteration with soft-deodorized olive oils. Food Control.

[40] Koulis G.A., Tsagkaris A.S., Aalizadeh R., Dasenaki M.E., Panagopoulou E.I., Drivelos S., Halagarda M., Georgiou C.A., Proestos C., Thomaidis N.S. (2021). Honey Phenolic Compound Profiling and Authenticity Assessment Using HRMS Targeted and Untargeted Metabolomics. Molecules.

[41] Koulis G.A., Tsagkaris A.S., Katsianou P.A., Gialouris P.-L.P., Martakos I., Stergiou F., Fiore A., Panagopoulou E.I., Karabournioti S., Baessmann C. (2022). Thorough Investigation of the Phenolic Profile of Reputable Greek Honey Varieties: Varietal Discrimination and Floral Markers Identification Using Liquid Chromatography—High-Resolution Mass Spectrometry. Molecules.

[42] Abdossi V., Kazemi M. (2016). Bioactivities of *Achillea millefolium* essential oil and its main terpenes from Iran. Int. J. Food Prop..

[43] Ferracane R., Graziani G., Gallo M., Fogliano V., Ritieni A. (2010). Metabolic profile of the bioactive compounds of burdock (*Arctium lappa*) seeds, roots and leaves. J. Pharm. Biomed. Anal..

[44] Petropoulos S.A., Fernandes Â., Tzortzakis N., Sokovic M., Ciric A., Barros L., Ferreira I.C.F.R. (2019). Bioactive compounds content and antimicrobial activities of wild edible *Asteraceae* species of the Mediterranean flora under commercial cultivation conditions. Food Res. Int..

[45] Lusa M.G., Martucci M.E.P., Loeuille B.F.P., Gobbo-Neto L., Appezzato-da-Glória B., Da Costa F.B. (2016). Characterization and evolution of secondary metabolites in Brazilian Vernonieae (Asteraceae) assessed by LC-MS fingerprinting. Bot. J. Linn. Soc..

[46] Burlec A.F., Arsene C., Gille E., Hăncianu M., Cioancă O. (2017). Ornamental Asteraceae species as new sources of secondary metabolites. Indian J Pharma Educ Res.

[47] Pljevljakušić D., Bigović D., Janković T., Jelačić S., Šavikin K. (2018). Sandy everlasting (*Helichrysum arenarium* (L.) Moench): Botanical, chemical and biological properties. Front. Plant Sci..

[48] Rajan L., Palaniswamy D., Mohankumar S.K. (2020). Targeting obesity with plant-derived pancreatic lipase inhibitors: A comprehensive review. Pharmacol. Res..

[49] Ak G., Zengin G., Ceylan R., Fawzi Mahomoodally M., Jugreet S., Mollica A., Stefanucci A. (2021). Chemical composition and biological activities of essential oils from *Calendula officinalis* L. flowers and leaves. Flavour Fragr. J..

[50] Butnariu M., Coradini C.Z. (2012). Evaluation of biologically active compounds from Calendula officinalis flowers using spectrophotometry. Chem. Cent. J..

[51] El Moghazy A.M., Darwish F.M., El Khayat E.S., Mohamed M.O., Wink M., El Readi M.Z. (2011). New Hexadecan-2-ol and 3-Hydroxymethyloctadecanoate with Hepatoprotective Activity and Cytotoxicity Activity from Grindelia camporum Greene (Asteraceae). Planta Med..

[52] Bijak M. (2017). Silybin, a major bioactive component of milk thistle (*Silybum marianum* L. Gaernt.)—Chemistry, bioavailability, and metabolism. Molecules.

[53] Corchete P. (2008). *Silybum marianum* (L.) Gaertn: The source of silymarin. Bioactive Molecules and Medicinal Plants.

[54] Tajmohammadi A., Razavi B.M., Hosseinzadeh H. (2018). Silybum marianum (milk thistle) and its main constituent, silymarin, as a potential therapeutic plant in metabolic syndrome: A review. Phyther. Res..

[55] Ali S., Zameer S., Yaqoob M. (2017). Ethnobotanical, phytochemical and pharmacological properties of *Galinsoga parviflora* (Asteraceae): A review. Trop. J. Pharm. Res..

[56] Ayaz F.A., Ozcan M., Kurt A., Karayigit B., Ozogul Y., Glew R., Ozogul F. (2017). Fatty acid composition and antioxidant capacity of cypselas in *Centaurea s.l.* taxa (Asteraceae, Cardueae) from NE Anatolia. S. Afr. J. Bot..

[57] Soetardjo S., Jong P.C., Noor A.M., Lachimanan Y.L., Sreenivasan S. (2007). Chemical composition and biological activity of the *Centipeda minima* (Asteraceae). Malays. J. Nutr..

[58] Kuczmannová A., Gál P., Varinská L., Treml J., Kováč I., Novotný M., Vasilenko T., Dall’Acqua S., Nagy M., Mučaji P. (2015). *Agrimonia eupatoria* L. and *Cynara cardunculus* L. water infusions: Phenolic profile and comparison of antioxidant activities. Molecules.

[59] Lin L.-Z., Harnly J.M. (2010). Identification of the phenolic components of chrysanthemum flower (*Chrysanthemum morifolium* Ramat). Food Chem..

[60] Salomon L., Lorenz P., Bunse M., Spring O., Stintzing F.C., Kammerer D.R. (2021). Comparison of the Phenolic Compound Profile and Antioxidant Potential of *Achillea atrata* L. and *Achillea millefolium* L.. Molecules.

[61] Palić R., Stojanović G., Ranđelović N., Ranđelović V., Veličković J. (2000). The fatty acids from plants of the genus Achillea. Facta Univ. Phys. Chem. Technol..

[62] Chen S., Liu J., Dong G., Zhang X., Liu Y., Sun W., Liu A. (2021). Flavonoids and caffeoylquinic acids in *Chrysanthemum morifolium* Ramat flowers: A potentially rich source of bioactive compounds. Food Chem..

