# Supplementary material for: The In Vitro Inhibitory Effect of Selected Asteraceae Plants on Pancreatic Lipase Followed by Phenolic Content Identification through Liquid Chromatography High Resolution Mass Spectrometry (LC-HRMS)
Source: Int J Mol Sci. 2022 Sep 23;23(19):11204. doi: 10.3390/ijms231911204 (PMC9569725; doi:10.3390/ijms231911204)
Supplement: Supplementary file 1 [file ijms-23-11204-s001.zip › ijms-1897157-supplementary/supp material_PNLIP assay_final_revisions_AT.pdf]

SUPPLEMENTARY MATERIALS

The In Vitro Inhibitory Effect of Selected Asteraceae Plants on Pancreatic Lipase Followed by Phenolic Content Identification through Liquid Chromatography High Resolution Mass Spectrometry (LC-HRMS)

A. S. Tsagkaris[a*], A. Louckova[a], T. Jaegerova[a], V. Tokarova[b], J. Hajslova[a]

a. Department of Food Analysis and Nutrition, Faculty of Food and Biochemical Technology, University of Chemistry and Technology Prague, Technická 5, 166 28 Prague 6 – Dejvice, Prague, Czech Republic
b. Department of Chemical Engineering, Faculty of Chemical Engineering, University of Chemistry and Technology Prague, Technická 5, 166 28 Prague 6 – Dejvice, Prague, Czech Republic

Corresponding authors: tsagkara@vscht.cz

S1. Supporting information on substrate selection

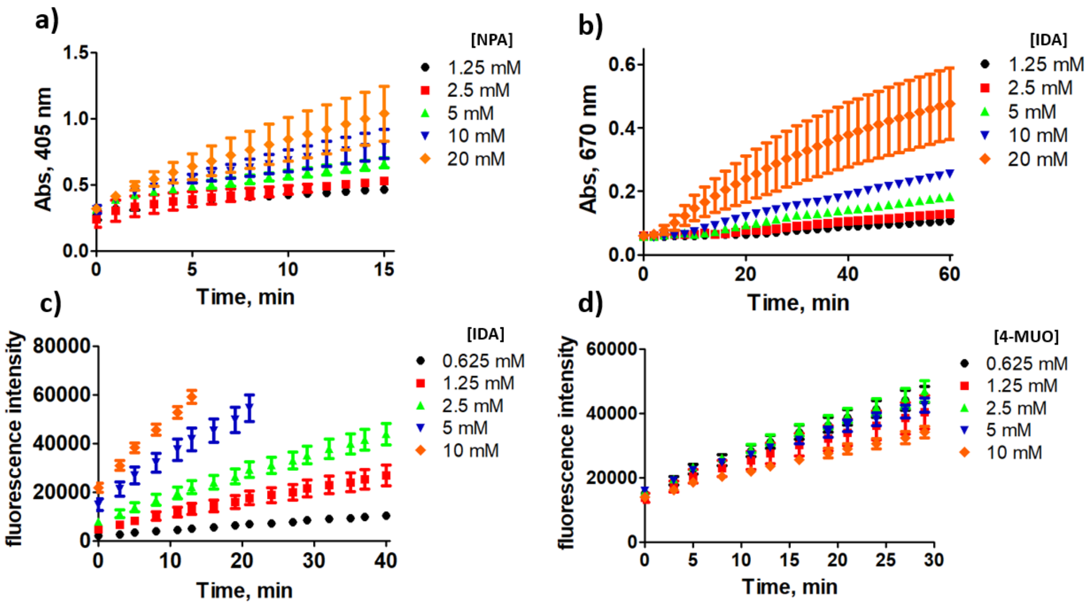

Figure S1. PNLIP assay signal development using a) NPA, b,c) IDA and d) 4-MUO as the substrate. PNLIP concentration was 1250 µg mL-1 in every case and each point represents the mean value (n=9) and the error bars represent the standard deviation in each case.

S2. Supporting information on assay optimisation

Table S1. Calculated Km and Vmax at different end-points using 1250 µg mL^{-1} PNLIP. The results are expressed as "mean (95% intervals)" (n=6 replicates per level).

end-point (min)	Km (mM)	Vmax (dAbs/min)
5	16 (9.6-22)	0.066 (0.054-0.078)
10	14 (6.2-23)	0.046 (0.035-0.058)
20	17 (4.6-29)	0.043 (0.029-0.058)
40	14 (8.1-19)	0.035 (0.029-0.041)
60	16 (10-22)	0.035 (0.029-0.041)

S3. Indicative XIC chromatograms

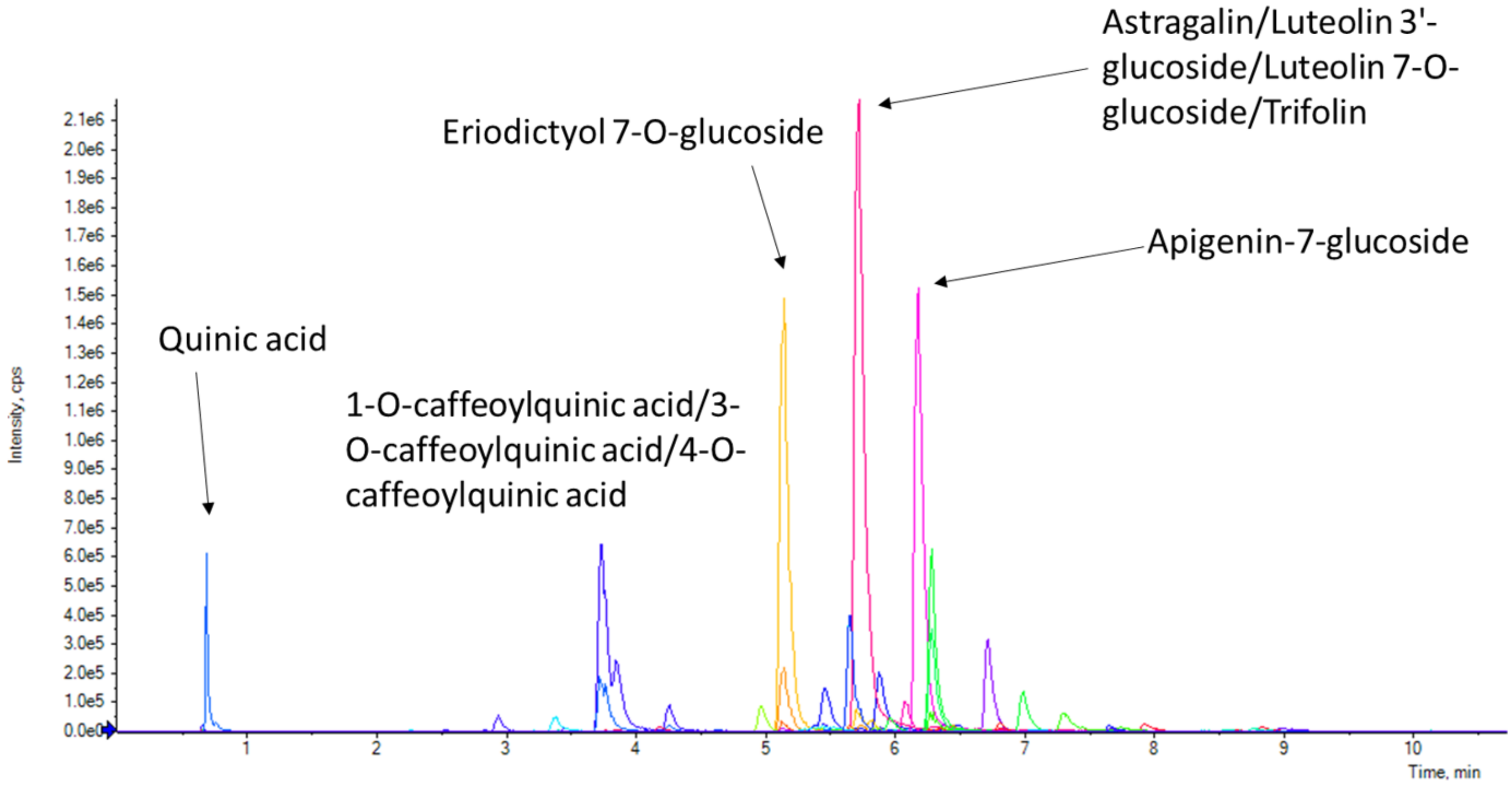

Figure S2. XIC chromatogram of *Chrysantemum morifolium* flower aqueous extract, ESI-.

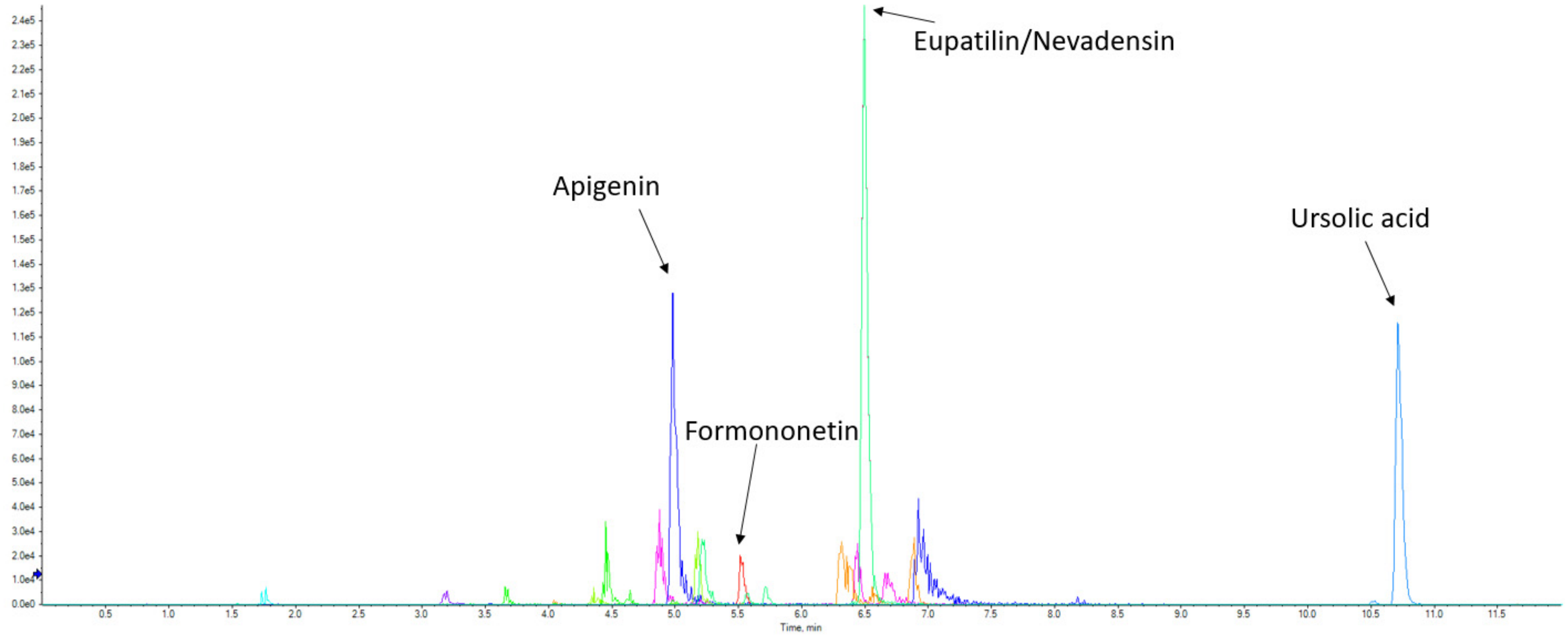

Figure S3. XIC chromatogram of *Achillea millefolium* leaves dichloromethane extract, ESI-.

S4. Enzyme assays protocols

PNLIP assay using NPA as the substrate

Eighty µL PNLIP (1250 µg mL $^{-1}$) were incubated with 10 µL of a sample for 15 min. Next, 10 µL of a NPA solution (in the range 1.25-20 mM) in DMSO were added and the absorbance was measured at 405 nm for 15 min.

PNLIP assay using IDA as the substrate

Eighty µL PNLIP (1250 µg mL $^{-1}$) were incubated with 10 µL of a sample for 15 min. Next, 10 µL of an IDA solution (in the range 1.25-20 mM) in DMSO were added and the absorbance was measured at 670 nm for 60 min. In the case of fluorescence monitoring, 10 µL of an IDA solution (in the range 0.625-10 mM) in DMSO were added and the fluorescent signal was monitored using the following wavelengths λexc= 395 nm and λem= 470 nm.

PNLIP assay using 4-MUO as the substrate

Eighty µL PNLIP (1250 µg mL $^{-1}$) were incubated with 10 µL of a sample for 15 min. Next, 10 µL of a 4-MUO solution (in the range 0.625-10 mM) in DMSO were added and the fluorescent signal was monitored using the following wavelengths λexc= 355 nm and λem= 460 nm.

S5. Tested plants

Table S2. The list of samples investigated for PNLIP inhibition potency.

Botanical Family	Species	Common name	Part of plant
Asteraceae	*Achillea millefolium*	Yarrow	Leaves
	Anthemis nobilis	Chamomile Herb	Flower
	Arctium lappa	Burdock Root	Root
	Artemisia abrotanum	Southernwood Herb	Leaf
	Artemisia absinthium	Wormwood	Aerial parts
	Artemisia annua	Sweet wormwood (Qing Hao)	Stem

Artemisia dracunculus	Tarragon Herb	Leaf
Artemisia vulgaris	Mugwort Herb	Aerial parts
Atractylodes macrocephala	Atractylodes	Rhizome
Calendula officinalis	Marigold flowers	Flower
Calendula officinalis	Marigold Petals	Petal
Cichorium intybus	Chicory Root	Root
Cnicus benedictus	Holythistle	Aerial parts
Cynara cardunculus	Artichoke	Leaf of stem
Eclipta alba	Bhringaraj Root	Root
Echinacea angustifolia	Echinacea Angustifolia Root	Root
Echinacea purpurea	Echinacea Purpurea Root	Root
Eupatorium perfoliatum	Boneset	Leaf
Eupatorium purpureum	Gravel root	Root
Grindelia camporum	Grindelia Herb	Aerial parts
Helianthus annuus	Sunflower seed	Seed
Hieracium pilosella	Mousear, hawkweed	Aerial parts
Chrysanthemum morifolium	Chrysanthemum flowers	Flower
Inula helenium	Elecampane Root	Root
Lactuca sativa	Lettuce	Leaf
Lactuca virosa	Wild Lettuce	Leaf
Matricaria recutita	Chamomile, German	Flower
Silybum marianum	Milkthistle Seed	Seed
Solidago virgaurea	Golden Rod	Aerial parts
Stevia rebaudiana	Stevia leaf	Leaf
Tanacetum parthenium	Feverfew Herb	Aerial parts
Tanacetum vulgare	Tansy Herb	Aerial parts
Taraxacum officinale	Dandelion Herb	Leaf
Taraxacum officinale	Dandelion Root	Root
Tussilago farfara	Coltsfoot	Aerial parts

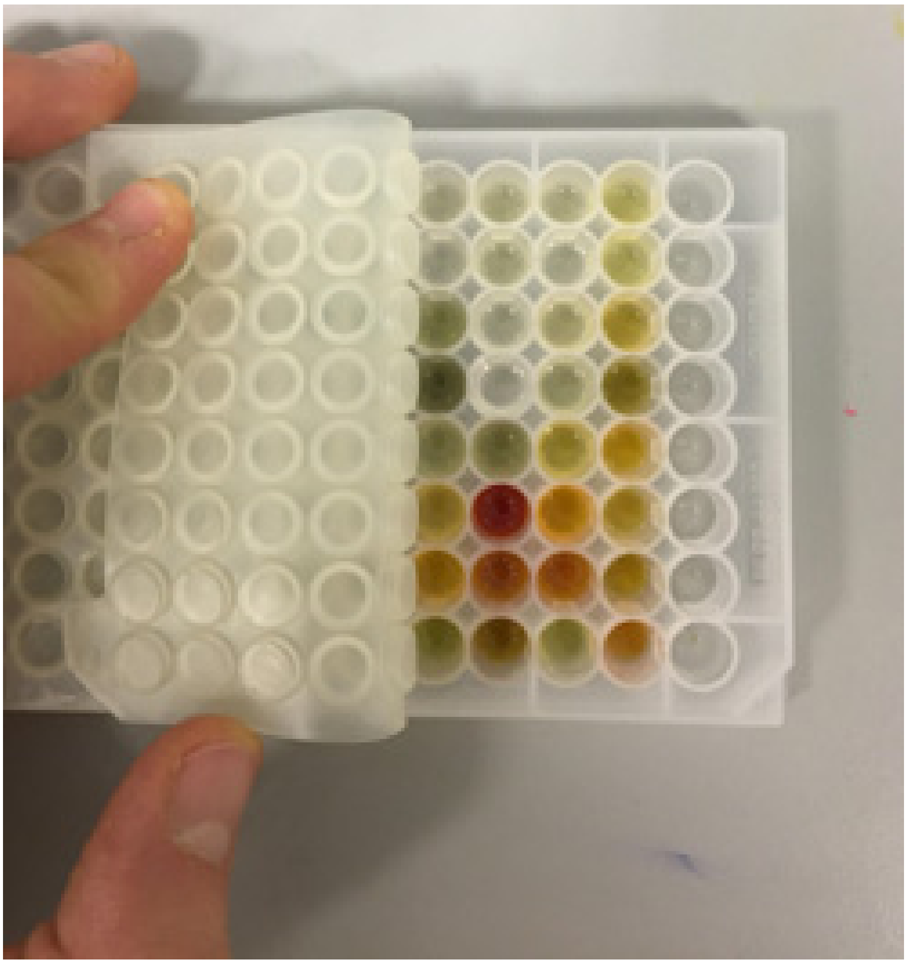

Figure S4. The microplate format in which the purchased extracts were stored

S6. Formulas for inhibition rate calculation

Raw data correction

1. Test sample raw absorbance was corrected by subtracting the raw data of the test blank sample and the raw data of substrate blank (both not containing any PNLIP solution) from the test sample raw data.

Corrected test sample Abs= $A_{test\ sample} - A_{test\ sample\ blank} - A_{substrate\ blank}$ (S1)

2. Positive control (orlistat) raw absorbance was corrected by subtracting the raw data of the substrate blank (not containing any PNLIP solution) from the positive control raw data.

Corrected positive control Abs= $A_{positive\ control} - A_{substrate\ blank}$ (S2)

3. Negative control-buffer blank: raw data of buffer blank was corrected by calculating raw data of buffer blank minus substrate blank.

Corrected negative control Abs= $A_{test\ buffer\ blank} - A_{test\ substrate\ blank}$ (S3)

Inhibition rate% using the blank corrected data

4. The inhibition rate% was based on the ratio of the corrected absorbance of a sample (test sample or positive control) divided by the corrected absorbance of the negative control.

Inhibition rate%= $1 - (A_{corrected\ sample} / A_{corrected\ negative\ control}) \times 100$ (S4)
